# Effect of Granodiorite Sand Content and Particle Size on the Mechanical and Thermal Performance of Metakaolin-Based Geopolymer Mortar

**DOI:** 10.1021/acsomega.6c01784

**Published:** 2026-06-17

**Authors:** Yildiz Yildirim, Gurkan Akarken, Gokçe Kayatepe, Ugur Cengiz

**Affiliations:** † Kale Ceramic R&D Department, Kale Group, 17430 Çan, Çanakkale, Türkiye; ‡ Department of Energy Resources and Management, Faculty of Engineering, Çanakkale Onsekiz Mart University, 17010 Çanakkale, Türkiye; § AFC Green Technologies R&D, Canakkale Technopark, Sarıcaeli, 17100 Çanakkale, Türkiye; ∥ Surface Science Research Laboratory, Department of Chemical Engineering, Faculty of Engineering, 52950 Çanakkale Onsekiz Mart University, 17010 Çanakkale, Türkiye

## Abstract

In this study, the coupled effects of granodiorite sand particle size and content on the mechanical and thermal performance of metakaolin-based geopolymer systems are systematically investigated using a holistic, geometry-sensitive approach. For block-type specimens compressive and flexural strengths, together with postheating mass loss after exposure to 300 °C, are evaluated to elucidate strength retention and thermal stability. The results demonstrate that granodiorite particle size plays a decisive role in governing the balance between mechanical integrity and thermal insulation. Coarse particles (0.5–1.0 mm) at 30 wt % content provided the highest mechanical performance, achieving a compressive strength of approximately 50–68 MPa depending on the skeletal structure formation. Thermal stability tests at 300 °C showed that incorporating granodiorite effectively controlled degradation, with mass loss ranging from 3.4% to 9.1%, where the BG1–20 composition outperformed the control sample’s 5.7% loss. For plate-type specimens, thermal insulation was assessed under 700 °C flame loading. Fine granodiorite particles (<0.25 mm) markedly improved insulation by reducing back-surface temperatures to as low as 93 °C, significantly lower than the 207 °C observed with coarse particles. Overall, the findings reveal an inverse yet predictable relationship between mechanical strength and thermal insulation, providing a rational framework for designing geopolymer blocks and plates tailored to application-specific requirements.

## Graphical abstract

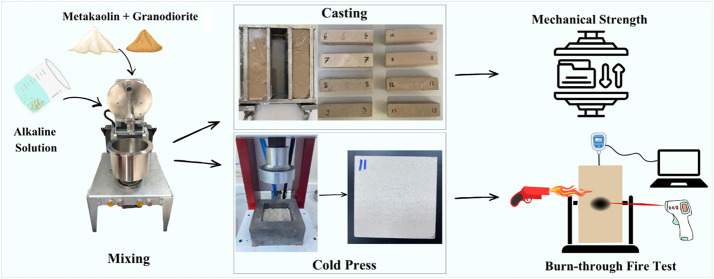

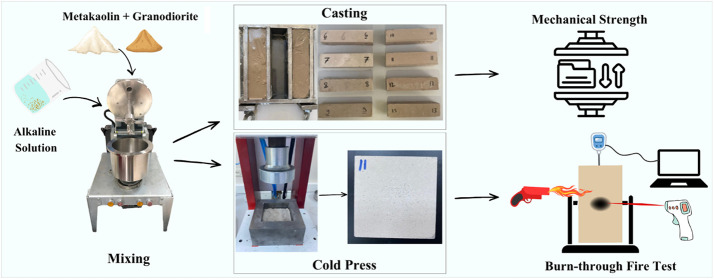

## 1 Introduction

The increasing energy consumption and associated carbon emissions in the construction sector have made it imperative to develop alternative binder systems with reduced environmental impact to replace conventional Portland cement–based materials.^1,2^ In particular, the high-temperature processing routes and CO_2_ emissions associated with limestone calcination have accelerated research into sustainable construction materials.^1–3^ In this context, geopolymer systems, which are characterized by a low carbon footprint and the ability to incorporate industrial byproducts or natural aluminosilicates, have emerged as a prominent research focus in recent years.^4–7^ Geopolymerization is a polycondensation process based on the dissolution of aluminosilicate precursors in alkaline activators, followed by the formation of a three-dimensional network structure characterized by Si–O–Al bonds.^4^ Unlike hydration-based Portland cement systems, this process results in the development of an amorphous or semicrystalline aluminosilicate gel phase.^8,9^ Highly reactive and high-purity aluminosilicate precursors such as metakaolin play a critical role in determining the mechanical and thermal performance of geopolymer binders.^10,11^ One of the most notable characteristics of geopolymers is their inherent resistance to elevated temperatures, which is closely associated with their silicate-based aluminosilicate network structure.^12,13^ The stable bonds formed between silicon and oxygen promote the formation of noncombustible, low-volatility, and thermally stable material systems, a behavior that has been experimentally confirmed in metakaolin-based geopolymer networks at elevated temperatures.^4,12,14–16^ Furthermore, Celik et al. highlighted that the inherent thermal resistance of metakaolin-based geopolymer composites is largely attributed to the stable microstructural continuity and the dense amorphous nature of the aluminosilicate gel, which restrict severe thermal damage at elevated temperatures.^17^

In addition to the binder chemistry, the aggregates and filler materials used in geopolymer systems play a decisive role in governing both mechanical and thermal performance.^6,18,19^ The incorporation of sand and mineral fillers significantly affects stress distribution, microstructural density, and porosity characteristics within the geopolymer matrix by altering particle packing efficiency, matrix–aggregate interfacial interactions, and pore structure continuity, which in turn govern the mechanical response of geopolymer composites.^18,20^ Previous studies have demonstrated that mineral fillers and aggregates can reduce shrinkage, enhance dimensional stability under elevated temperatures, and significantly modify the mechanical response of geopolymer systems by altering microstructural densification and porosity characteristics.^12,21–24^ For instance, Kuenzel et al. investigated the influence of sand addition on the mechanical properties of metakaolin-based geopolymers, demonstrating that an optimal sand content minimizes drying shrinkage and significantly increases compressive strength, whereas excessive sand addition leads to a weaker matrix due to insufficient binder coverage.^25^ Furthermore, Zhang et al. evaluated the thermal behavior of geopolymer mortars and reported that while the specimens retained substantial residual compressive strength after exposure to elevated temperatures, the extent of thermal damage and microcracking was highly dependent on the aggregate type and the resulting pore structure.^26^ Additionally, Shaikh and Haque demonstrated that the inclusion of fine silica sand significantly improved the residual compressive strength of geopolymers exposed to elevated temperatures, as the sand particles provided a stable load-bearing framework that resisted thermal degradation.^27^ Previous investigations have further shown that alkali activation can promote the formation of dense and complex gel networks through hydration and polymerization mechanisms, significantly influencing microstructural development and performance evolution in geopolymer-based systems.^28^

In particular, particle size distribution has been identified as a key microstructural parameter governing both the mechanical response and thermal performance of geopolymer composites by influencing densification and porosity evolution under thermal exposure.^29,30^ Fine-grained fillers and aggregates tend to promote higher microporosity and increased air entrapment within the geopolymer matrix, which effectively limits heat transfer and reduces thermal conductivity, albeit at the expense of mechanical strength.^12,21,29^ In this context, Kong et al. compared the high-temperature performance of different geopolymer matrices and revealed that thermal shrinkage and the thermo-mechanical mismatch between the aluminosilicate gel and the aggregate particles are the primary mechanisms governing strength degradation and microstructural stability under thermal exposure.^31^ As Arslan et al. pointed out, while the evaporation of free and chemically bound water at high temperatures causes internal microstructural damage and mass loss, optimizing the solid filler content can successfully mitigate this degradation and maintain the dimensional stability of the geopolymer matrix.^32^ In contrast, coarse aggregates promote the formation of a denser, more continuous, load-bearing skeletal structure, leading to enhanced compressive strength. However, it has also been emphasized that increased porosity may limit mechanical strength while improving thermal insulation performance, resulting in an inverse yet predictable relationship between mechanical and thermal properties.^21,23,33^

Granodiorite, owing to its high silica content, quartz–feldspar-dominated mineralogical composition, and inherently favorable mechanical characteristics, constitutes a promising natural aggregate candidate for geopolymer-based mortars. Previous studies have demonstrated that silica-rich fine aggregates and sands can significantly influence geopolymer reaction kinetics, matrix densification, and interfacial bonding by modifying particle packing efficiency and pore size distribution.^27,34^ In geopolymer systems, variations in aggregate particle size and dosage govern not only compressive and flexural strength development but also transport-related properties such as permeability and heat transfer through their effect on pore connectivity and interfacial transition zones.^35,36^

Although the role of sand type and grading in geopolymer mortars and concretes has been broadly acknowledged, existing research predominantly focuses on generic siliceous sands or industrial byproducts, with limited attention paid to granodiorite-specific systems.^37^ More critically, systematic and comparative investigations addressing the coupled influence of granodiorite particle size and content on both mechanical performance and thermal behavior—particularly across different product geometries such as blocks and plates—remain notably scarce. This lack of comparative, geometry-sensitive assessment restricts a comprehensive understanding of structure–property relationships in granodiorite-containing geopolymer mortars and highlights the need for targeted experimental studies in this area.

In this study, the coupled effects of granodiorite sand particle size and content on the mechanical and thermal performance of metakaolin-based geopolymer systems are systematically investigated using a holistic, geometry-sensitive approach. The primary purpose of this research is to bridge the existing knowledge gap regarding the thermomechanical optimization of granodiorite-incorporated geopolymers, thereby facilitating their practical and large-scale structural utilization. For block-type specimens, compressive and flexural strengths, together with postheating mass loss after exposure to 300 °C, are evaluated to elucidate strength retention and thermal stability. For plate-type specimens, thermal insulation performance is quantitatively assessed by monitoring back-surface temperature evolution under direct flame exposure at 700 °C. Furthermore, to comprehensively understand the porosity and durability characteristics, the total water absorption capacity of the composites is also determined. This integrated experimental framework enables a clear elucidation of particle size–dependent microstructural effects governing the trade-off between mechanical integrity and thermal insulation. The importance of this study lies in its potential to provide the construction sector with eco-friendly, fire-resistant, and mechanically robust alternative building materials. By identifying the optimal matrix combinations, the outcomes of this study are expected to establish a rational basis for optimizing granodiorite particle size and dosage in geopolymer blocks and plates designed for application-specific performance requirements, ultimately contributing to sustainable development and energy-efficient building designs.

## 2 Materials Method

### 2.1 Materials

Metakaolin (MK) was used as the primary aluminosilicate precursor and was obtained from AVS Mineral (Istanbul, Türkiye). Prior to specimen preparation, MK was oven-dried at 100 °C for 3 h to eliminate residual moisture and ensure reproducible mixing conditions. Altered granodiorite sand (GS), used as the aggregate phase, was sourced from local granodiorite deposits in Çanakkale Province, Türkiye. The sand was classified into three particle size fractions: <0.25 mm, 0.25–0.5 mm, and 0.5–1.0 mm. Alkali activation was achieved using sodium hydroxide (NaOH) flakes with a purity of ≥99.0%, sourced from Interlab (Istanbul, Türkiye), and a commercial sodium silicate (Na_2_SiO_3_) solution provided by Sodel Chemical Industry Inc. (Denizli, Türkiye). The sodium silicate activator had a SiO_2_/Na_2_O molar ratio of 2, a density of 1.5 g/cm^3^, and consisted of 28.2 wt % SiO_2_, 13.5 wt % Na_2_O, and 58 wt % H_2_O. Prismatic iron molds with dimensions of 4 cm × 4 cm × 16 cm were designed and fabricated locally in accordance with standard geometrical requirements for flexural strength testing. In addition to these specimens, plate-type samples with dimensions of 10 cm × 10 cm × 1 cm were produced by the cold-press technique.

### 2.2 Preparation of Granodiorite-Modified Geopolymer Mortars

This study examines the influence of granodiorite sand incorporation on the mechanical and thermal performance of metakaolin-based geopolymer mortars. Granodiorite sand was introduced into the geopolymer matrix at mass fractions of 10%, 20%, and 30%, using three particle size ranges (<0.25 mm, 0.25–0.5 mm, and 0.5–1.0 mm). For block-type specimens produced by conventional casting, a total of 12 sand-containing compositions were designed by systematically varying sand content and particle size, together with a sand-free reference geopolymer (BG0).

A concise sample coding system was adopted to ensure consistent identification of specimens throughout the study. The notation G0, G1, G2, and G3 represents geopolymer mixtures containing no sand, <0.25 mm, 0.25–0.5 mm, and 0.5–1.0 mm granodiorite sand, respectively, while the numerical suffix denotes the sand content by mass percentage. Block-type specimens (4 cm × 4 cm × 16 cm) produced by casting are identified with the prefix “B”, whereas plate-type specimens (10 cm × 10 cm × 1 cm) fabricated by cold pressing are denoted by the prefix “P”. For plate specimens, sand contents of 20% and 30% were selected for each particle size range, along with a sand-free reference plate (PG0), resulting in seven plate-type compositions. The complete mix configurations and specimen codes are summarized in Table 1.

**Table 1:** Sample Codes and Mix Configurations of Granodiorite Modified Geopolymer Specimens^a^

sample	GPS (mm)	GS (wt %)	GS/M (%)	production method
BG0		0	0	Casting
BG1–10	<0.25	10	17.7	Casting
BG1–20	<0.25	20	32.7	Casting
BG1–30	<0.25	30	45.4	Casting
BG2–10	0.25–0.5	10	17.7	Casting
BG2–20	0.25–0.5	20	32.7	Casting
BG2–30	0.25–0.5	30	45.4	Casting
BG3–10	0.5–1.0	10	17.7	Casting
BG3–20	0.5–1.0	20	32.7	Casting
BG3–30	0.5–1.0	30	45.4	Casting
PG0		0	0	Cold Press
PG1–20	<0.25	20	32.7	Cold Press
PG1–30	<0.25	30	45.4	Cold Press
PG2–20	0.25–0.5	20	32.7	Cold press
PG2–30	0.25–0.5	30	45.4	Cold press
PG3–20	0.5–1.0	20	32.7	Cold press
PG3–30	0.5–1.0	30	45.4	Cold press

a GS denotes altered granodiorite sand, and GPS represents the particle size of altered granodiorite sand. GS (wt %) refers to the mass percentage of altered granodiorite sand relative to the total mixture. GS/M (%) denotes the proportion of altered granodiorite sand within the binder system and is calculated as GS/(GS + metakaolin) × 100.

The alkaline activator system was prepared by dissolving sodium hydroxide (in flake form) directly into sodium silicate solutions, maintaining a fixed **Na**_**2**_ **SiO**_**3**_ **/NaOH molar ratio of 3.2**. The liquid-to-solid (L/S) ratio was adjusted according to the specific production method to ensure optimal consistency: for cold-pressed plate-type specimens, the L/S ratio was maintained at 0.30 (30 wt % liquid and 70 wt % solid), while for cast block-type specimens, it was increased to 2.61 (approximately 72.3 wt % liquid and 27.7 wt % solid) to achieve adequate flowability.

Beyond these physical ratios, the chemical design of the geopolymerization process was governed by fixed target molar ratios derived from the oxide compositions of the raw materials and the activator system. Accordingly, the mixtures were formulated with SiO_2_/Al_2_O_3_ = 4, (Na_2_O + K_2_O)/SiO_2_ = 0.35, H_2_O/(Na_2_O + K_2_O) = 18, and (Na_2_O + K_2_O)/Al_2_O_3_ in the range of 1.0–1.5. By keeping both the activator composition and these chemical ratios constant, a systematic evaluation of the effects of granodiorite particle size and content was ensured across all experimental groups.

The preparation and shaping procedures of the geopolymer mortars are schematically illustrated in Figure 1. Following the preparation of the alkaline activator as described above, the solution was mechanically stirred for approximately 15 min at room temperature to ensure complete homogenization. In parallel, metakaolin and granodiorite sand were dry-mixed to achieve a uniform particle distribution. The activator solution was then gradually added to the solid mixture and mixed until a homogeneous geopolymer paste was obtained.

**Figure 1:**
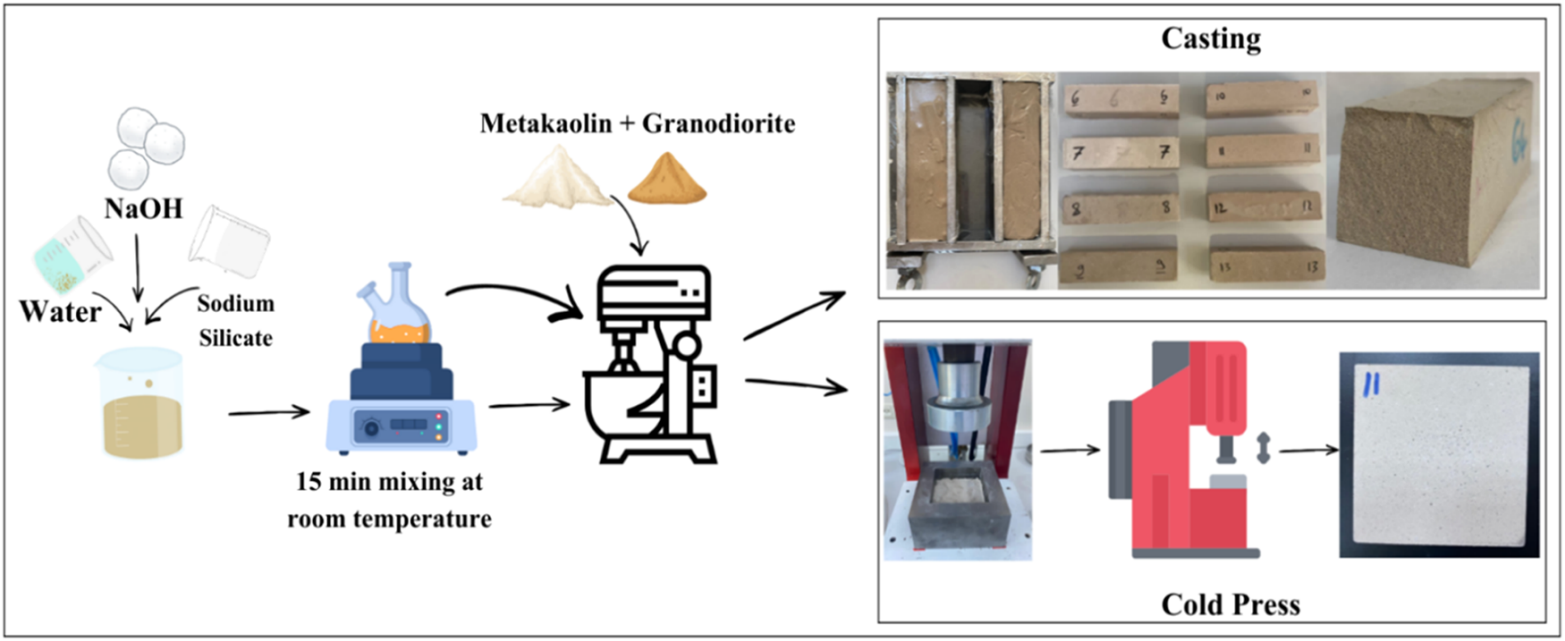
Schematic illustration of the preparation and shaping procedures of granodiorite modified geopolymer specimens.

For block-type specimens, the fresh geopolymer mixture was poured into prismatic steel molds (4 cm × 4 cm × 16 cm) and shaped by conventional casting. For plate-type specimens, the geopolymer mixture—containing a fixed alkaline solution content of 30 wt % relative to the total mass—was shaped using a cold-press technique. The mixed material was compacted under a hydraulic pressure of 400 kg/cm^2^ for 2 min to produce dense plates with dimensions of 10 cm × 10 cm × 1 cm. Following shaping, all specimens were cured under ambient laboratory conditions prior to mechanical, thermal, and flame exposure testing.

### 2.4 Characterization and Performance Evaluation Methods

The chemical compositions of the metakaolin and geopolymer specimens were determined by X-ray fluorescence (XRF) analysis (Panalytical Axios Max) (Table 2). In this table, the oxide and elemental weight percentages of the MK precursor are presented. The results reveal that MK is predominantly composed of **SiO**_**2**_ **(53.00 wt %)** and **Al**_**2**_ **O**_**3**_ **(43.80 wt %)**, which are the essential aluminosilicate sources required for the geopolymerization process. The presence of minor constituents such as **TiO**_**2**_ **(1.70 wt %)** and **Fe**_**2**_ **O**_**3**_ **(0.43 wt %)** is also observed, while alkaline and earth alkaline oxides like **CaO, MgO, Na**_**2**_ **O, and K**_**2**_ **O** remain at very low levels (all below 0.3 wt %). This high purity and high reactive oxide content confirm that the metakaolin used in this study is a suitable primary aluminosilicate precursor for forming a stable three-dimensional geopolymer network.

**Table 2:** Chemical Composition (wt %) of MK

compound	weight %	element	weight %
**SiO**_**2**_	53.00	**Si**	24.77
**Al**_**2**_ **O**_**3**_	43.80	**Al**	23.18
**TiO**_**2**_	1.70	**Ti**	1.02
**Fe**_**2**_ **O**_**3**_	0.43	**Fe**	0.30
**CaO**	0.02	**Ca**	0.014
**MgO**	0.03	**Mg**	0.018
**Na**_**2**_ **O**	0.23	**Na**	0.171
**K**_**2**_ **O**	0.19	**K**	0.158

The mineralogical phases were identified using X-ray diffraction (XRD) analysis (X’Pert Pro MPD, Cu Kα radiation), while the chemical bonding characteristics were analyzed by Fourier Transform Infrared (FTIR) spectroscopy using an ATR-equipped spectrometer (Bruker Tensor II). The surface morphologies of the specimens were examined by scanning electron microscopy (SEM) using a JEOL electron microscope.

The flexural strength of the block-type geopolymer specimens (4 cm × 4 cm × 16 cm) was measured using a Gabrielli S.R.L. CRS strength testing device in accordance with TS EN ISO 10545–4. Mechanical tests were performed on five specimens for each composition. The reported values represent the mean of these measurements, and the variability is expressed as standard deviation. For flame resistance evaluation, the prepared 10 cm × 10 cm geopolymer plates were fixed to the experimental setup and exposed to high-temperature flame heating on the front surface, with the surface temperature increased to approximately 700 °C under controlled conditions. The thermal loading configuration was designed to simulate burn-through test conditions reported by Sarazin et al.^38^ During the test, temperature evolution on both the front and back surfaces was continuously monitored, and the temperature difference across the plate thickness was recorded using an ORDEL UDL 100 universal data logger (Figure S1).

The thermal stability of the geopolymer blocks was further investigated by exposure to elevated temperature in a high-temperature muffle furnace (Magma Therm MT1107-B2). The specimens were heated to 300 °C at a constant heating rate of 5 °C/min and held isothermally for 1 h. After cooling to room temperature inside the furnace, mass variation and mechanical strength were evaluated before and after thermal exposure to assess the effect of elevated temperature on material performance (Figure S2). In order to systematically evaluate the porosity and moisture-related durability characteristics of the geopolymer mortars, the total water absorption capacity of the specimens was also determined using a progressive immersion method.^39^ The water absorption behavior of both granodiorite sand fractions and selected geopolymer specimens (BG1–30, BG2–30, and BG3–30) was determined to evaluate their pore-related characteristics. Granodiorite sand was classified into three discrete particle size ranges (<0.25 mm, 0.25–0.5 mm, and 0.5–1.0 mm) by sieve analysis, and the measurements were performed separately for each fraction. Prior to testing, the samples were oven-dried at 105 °C until constant mass was achieved and then cooled to room temperature (Figure S3A). The dried samples were subsequently immersed in water for 24 h to ensure full saturation (Figure S3B). After removal from water, excess surface moisture was carefully eliminated to obtain a saturated surface-dry condition, and the saturated mass was recorded. Water absorption values were calculated based on the mass difference between oven-dried and saturated conditions. The procedure was carried out in accordance with ASTM C128 (ASTM International, 2022),^40^ which is widely used for determining the water absorption characteristics of fine aggregates and porous construction materials.

## 3 Results and Discussion

### 3.1 Structural and Microstructural Characterization of Metakaolin and Geopolymer Matrix

The FTIR spectra of the metakaolin (M) and geopolymer (BG1–10) samples are presented in Figure 2.

**Figure 2:**
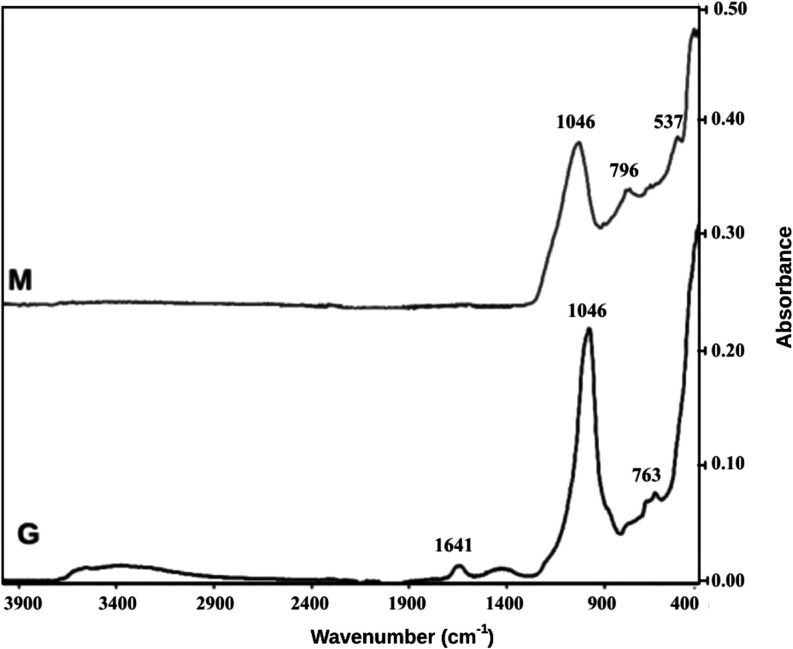
FTIR spectrum of Metakaolin (M) and its Geopolymer (G) forms.

The 1200–400 cm^–1^ region, reflecting the characteristic bond vibrations of aluminosilicate structures, constitutes the focus of the FTIR evaluation for all samples.^4^ In the FTIR spectrum of the metakaolin precursor, the structural −OH bands typically observed in the 3750–3500 cm^–1^ range are notably absent, confirming the successful dehydroxylation of kaolinite.^1^ The bands observed at approximately 1023 and 740 cm^–1^ are attributed to the internal stretching vibrations of Si–O–Si and Si–O–Al bonds. In addition, the bands at 798–800 cm^–1^ are associated with Al–O stretching vibrations in tetrahedral units, while the band at 914 cm^–1^ is specifically assigned to the asymmetric vibration of the amorphous content within the system.^41,42^

The FTIR spectra of the geopolymer mortars (e.g., BG0) exhibit pronounced band shifts and the emergence of new signals compared to metakaolin.^4^ As a result of the geopolymerization process, the intensity of the strong Al–O and Si–O bending vibrations near 790 cm^–1^ is significantly reduced, whereas new bands near 856 cm^–1^, indicative of tetrahedrally coordinated AlO_4_ units, become evident.^4^ The strong and broad bands detected in the 970–1068 cm^–1^ range are characteristic of asymmetric stretching vibrations of Si–O–Si and/or Al–O–Si bonds in the geopolymer network.^4^ The shift of these bands to approximately 80–90 cm^–1^ lower wavenumbers relative to metakaolin primarily reflects the significantly increased vitreous (amorphous) content formed during the alkaline activation.^9,43^ This prominent shift is driven by the structural transition into a highly disordered glassy network, alongside changes in the chemical environment of Si–O bonds caused by the substitution of SiO_4_ units by AlO_4_^–^ units within the aluminosilicate framework.^4,9^

In particular, the broad band at approximately 979–980 cm^–1^ serves as a characteristic indicator of asymmetric Si–O and Al–O vibrations in [(Si,Al)­O_4_] tetrahedra. Regarding the influence of the aggregate, the peak at 512 cm^–1^ represents the bending vibration of Si–O from the incorporated granodiorite sand, while the broad band in the 1080–1100 cm^–1^ region corresponds to the nonsolubilized silica of the added sand.^44^ Furthermore, the broad region at 3600–3000 cm^–1^ corresponds to water molecules retained within the structure, while the band at 1430–1440 cm^–1^ is assigned to the stretching vibrations of O–C bonds, indicating atmospheric carbonation and the formation of carbonate groups within the matrix.^45,46^ At lower wavenumbers, the 730–662 cm^–1^ range is attributed to symmetric atomic vibrations, and the 458–425 cm^–1^ range corresponds to Si­(Al)–O deformation vibrations.^7^ Overall, the FTIR results demonstrate that the amorphous structure of metakaolin reorganizes into a three-dimensional geopolymer network characterized by Si–O–Al linkages. The XRD patterns of the metakaolin (M) and metakaolin-based geopolymer (G) samples are shown in Figure 3.

**Figure 3:**
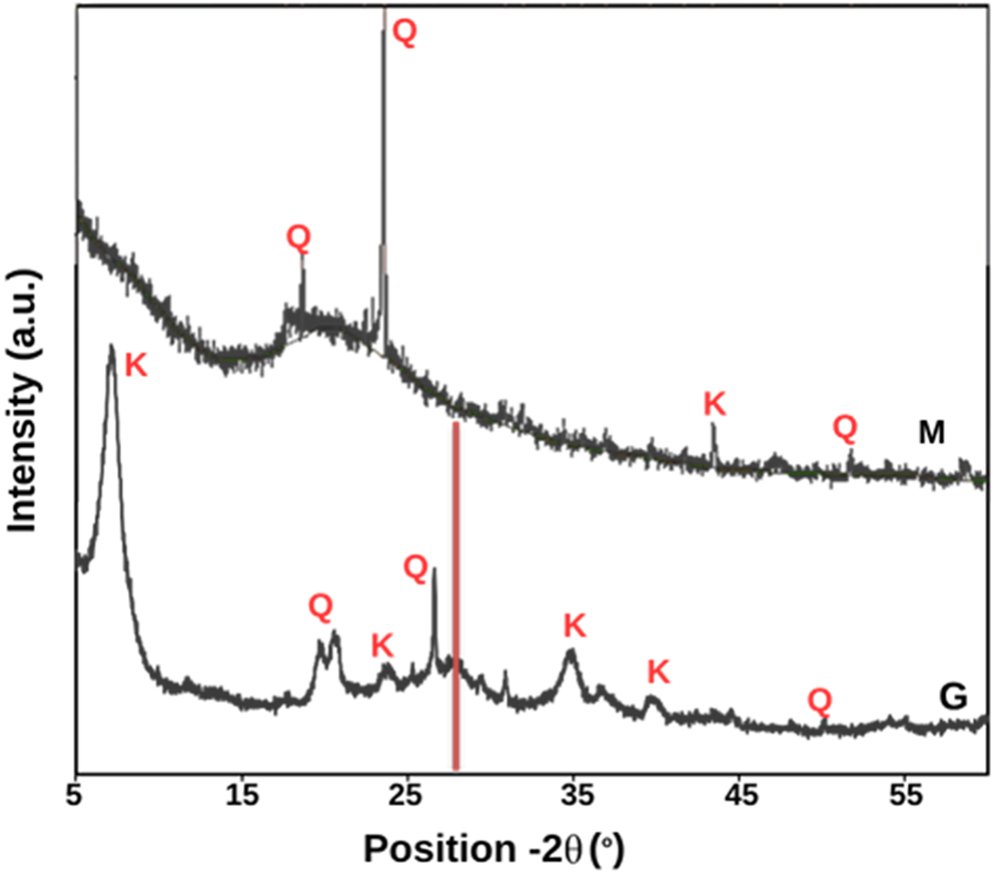
XRD spectrum of Metakaolen (M) and its Geopolymer (G) forms.

The XRD patterns of the geopolymer specimens exhibit a broad diffuse scattering halo, or amorphous hump, in the range of 17°–35° (2θ), which confirms the formation of a disordered aluminosilicate gel phase following the alkaline activation of metakaolin.^47,48^ This broad amorphous feature, centering at approximately 27° (2θ), indicates that the metakaolin precursor dissolves and reorganizes during the activation process, leading to the development of a stable geopolymer network.^4^ Based on the areas calculated from the XRD patterns, the sample was determined to consist of 61.91% amorphous phase and 38.09% crystalline phase. This high amorphous content is primarily associated with the geopolymeric aluminosilicate gel, which serves as the primary binding matrix of the material. The crystalline phases identified in the diffractogram are mainly attributed to residual quartz (Q) and partially reacted or unreacted kaolinite (K) originating from the granodiorite sand and metakaolin precursor.^49^ The persistence of quartz peaks indicates its inert behavior during geopolymerization, while the presence of kaolinite reflections suggests incomplete dissolution of the precursor phases.

The SEM images of the metakaolin sample (Figure 4) indicate a heterogeneous particle morphology. The presence of coarse, angular, and compact particles together with fine, flaky/plate-like, and agglomerated structures reflects the mineralogical diversity of metakaolin. Based on these morphological features, the angular and dense particles may be associated with quartz-rich regions, whereas the finer and platy structures are likely related to Illite-derived clay phases. However, it should be emphasized that this interpretation does not represent a direct phase identification but rather a morphology-based assessment supported by XRD and FTIR findings. The morphological variations observed in the SEM images are consistent with the crystalline–amorphous phase distribution determined by XRD and indicate that metakaolin possesses a predominantly amorphous structure containing minor crystalline remnants.

**Figure 4:**
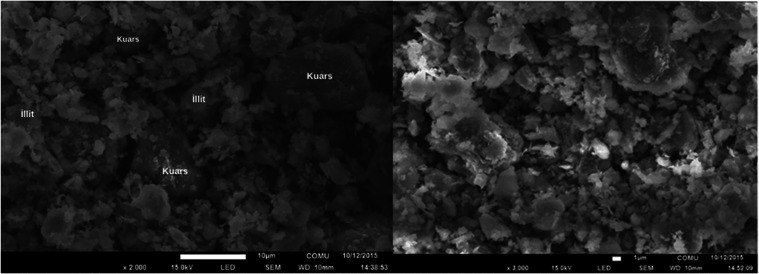
SEM images of Metakaolen particles.

SEM micrographs of the geopolymer systems without (BG0) and with granodiorite incorporation (BG2–30) are presented to elucidate the influence of aggregate addition on microstructural development (Figure 5). The BG0 sample exhibits a relatively homogeneous and continuous geopolymer matrix characterized by gel-like and layered structures, indicating a well-developed aluminosilicate network with limited structural discontinuities. In contrast, the BG2–30 sample, which was selected as a representative composition due to its intermediate particle size and highest granodiorite content, reveals a markedly different microstructure. Angular and dense granodiorite particles are clearly embedded within the geopolymer matrix, leading to a more heterogeneous structure. The presence of these particles disrupts matrix continuity and introduces distinct interfacial regions between the aggregate and the surrounding geopolymer gel.

**Figure 5:**
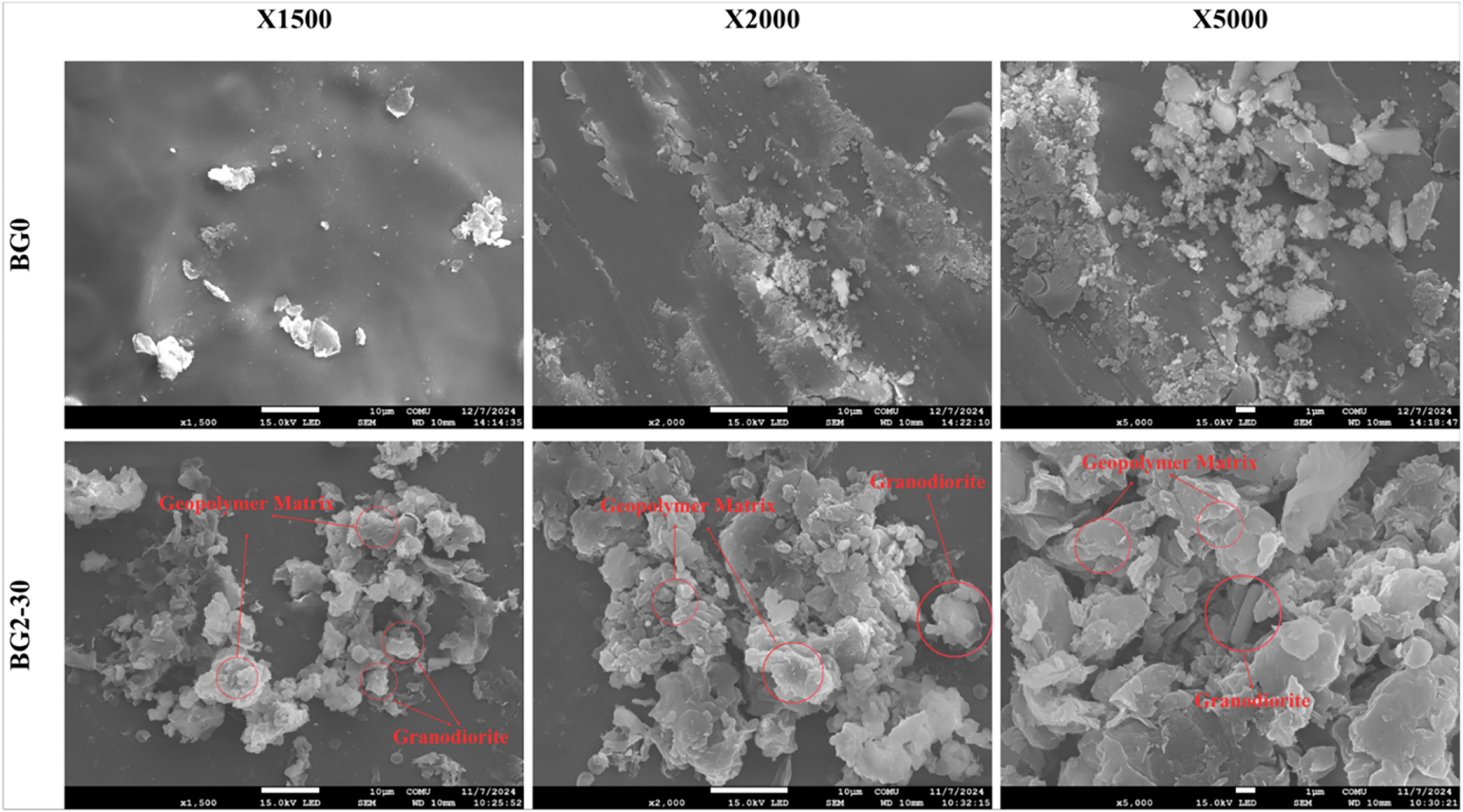
SEM micrographs of geopolymer systems without (BG0) and with granodiorite incorporation (BG2–30) at comparable magnifications.

Furthermore, localized interparticle voids and microstructural discontinuities are observed around the granodiorite inclusions, indicating a less compact packing arrangement compared to the sand-free system. These features are consistent with the experimentally observed decrease in density and increase in water absorption with increasing particle size. At the same time, the incorporation of rigid granodiorite particles contributes to load-bearing effects, which explains the improvement in mechanical strength observed for selected compositions. Overall, the SEM analysis demonstrates that the incorporation of granodiorite significantly alters the microstructural organization of the geopolymer matrix, establishing a direct link between particle size, porosity, and mechanical performance.

### 3.2 Effect of Granodiorite Sand Content and Particle Size on Mechanical Properties

Figure 6(a) illustrates the variation in compressive strength of block-type (BG-coded) geopolymer specimens, while Figure 6(b) presents the corresponding three-point flexural strength as a function of granodiorite sand content and particle size (GPS). The sand-free reference specimen (BG0) exhibits a compressive strength of approximately 40.94 MPa and a flexural strength of ∼4.9 MPa. With the incorporation of granodiorite, both mechanical properties show pronounced changes depending on sand content and particle size.

**Figure 6:**
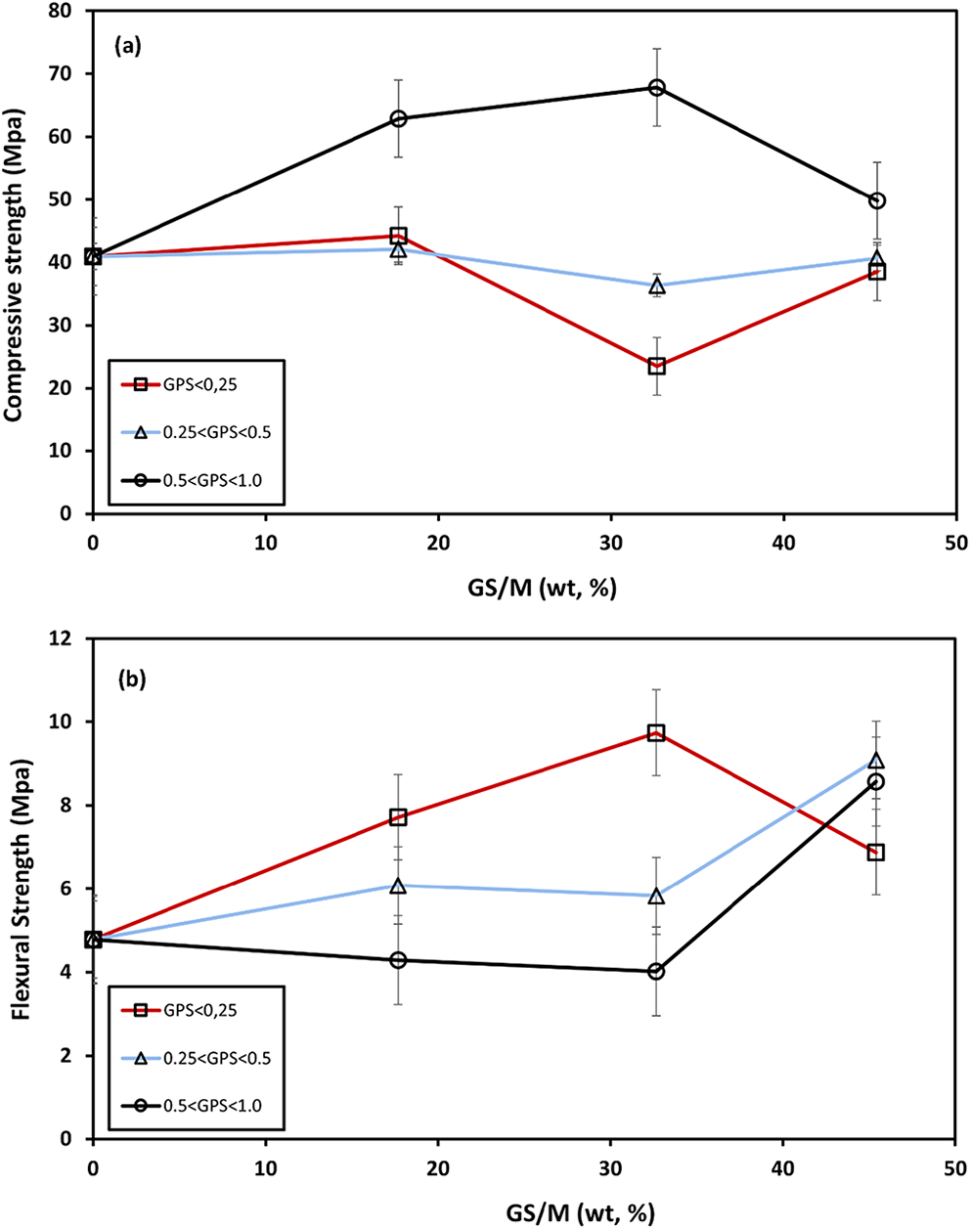
Effect of granodiorite content and particle size (GPS) on the compressive (a) and flexural (b) strengths of block-type geopolymer mortars. GS/M (%) represents the percentage of altered granodiorite sand relative to the total binder content. Error bars represent the standard deviation of five independent measurements.

As shown in Figure 6(a), compressive strength is strongly influenced by particle size, particularly for the coarse fraction (0.5 mm < GPS < 1.0 mm). According to Table 1, the highest sand incorporation corresponds to 30 wt.% GS (GS/M = 45.4%). For the coarse fraction, increasing granodiorite content from 10 to 30 wt.% (GS/M: 17.7–45.4%) results in a clear strength enhancement, with a maximum around 30 wt.%, followed by a reduction compared to the peak trend when the matrix continuity is compromised.

In contrast, finer particle size ranges (GPS < 0.25 mm and 0.25 mm < GPS < 0.5 mm) exhibit more moderate and comparable behavior, showing limited strength improvement and noticeable fluctuations, particularly at intermediate contents (20 wt.%, GS/M = 32.7%).

The flexural strength results (Figure 6(b)) demonstrate a different sensitivity to granodiorite incorporation. Fine particles (GPS < 0.25 mm) promote flexural strength up to 30 wt.% (GS/M = 45.4%), beyond which no further increase is observed within the studied range. Intermediate particles (0.25–0.5 mm) show their highest flexural strength at 30 wt.% (GS/M = 45.4%). For the coarsest fraction (0.5–1.0 mm), flexural strength remains relatively low at 10–20 wt.% (GS/M: 17.7–32.7%) but increases markedly at 30 wt.% (GS/M = 45.4%), indicating a more pronounced contribution to crack resistance at higher binder-replaced sand ratios.

Taken together, these results indicate that the role of granodiorite sand in geopolymer systems is strongly dependent on the type of mechanical loading, which explains the divergent correlation between compressive and flexural strengths. Under compressive loading, coarse particles at moderate contents act as a load-bearing skeleton, enhancing stress distribution within the matrix. Furthermore, fine granodiorite particles act as a microfiller that reduces the pore volume and densifies the matrix, contributing to compressive strength up to an optimal threshold;^27^ however, excessive sand addition limits the binder continuity and creates weak zones. Conversely, flexural performance is governed primarily by crack initiation and propagation mechanisms. Previous studies emphasize that flexural strength is inherently more sensitive to internal defects, porosity, and microcracks than compressive strength.^32,50^ Therefore, the flexural behavior of the mortars in this study is heavily dictated by the Interfacial Transition Zone (ITZ) between the granodiorite particles and the geopolymer gel. Strong particle–matrix interfacial bonding, crack-bridging, and mechanical interlocking effects, which are highly dependent on the aggregate morphology and surface adhesion,^34^ become dominant. Accordingly, coarse particles are more effective at higher contents for flexural resistance due to their higher mechanical interlocking capacity, whereas finer particles provide a more balanced response at intermediate contents.

Following this mechanical evaluation under ambient conditions, the thermal stability of the geopolymer blocks was assessed through controlled exposure at 300 °C. The specimens were heated at a constant rate of 5 °C min^–1^ and held at the target temperature for 1 h to simulate moderate thermal exposure while minimizing thermal shock effects.^51^ The resulting mass variations are summarized in Table 3.

**Table 3:** Mass Variation and Mass Loss of Geopolymer Specimens after Exposure to 300°C

sample	initial mass (g)	mass after heating (g)	mass loss (g)	mass loss (%)
BG0	130.8	123.3	7.5	5.7
BG1–20	205.9	199.0	6.9	3.4
BG1–30	212.2	200.4	11.8	5.8
BG2–20	210.6	194.5	16.1	7.6
BG2–30	208.5	192.3	16.2	7.8
BG3–20	211.6	193.0	18.6	8.8
BG3–30	211.4	192.1	19.3	9.1

The mass loss results are summarized in Table 3. All mixtures exhibited a measurable reduction in mass after thermal treatment. The calculated mass loss percentages ranged from 3.371% to 9.140%. The lowest mass loss was observed for the BG1–20 specimen, whereas the highest mass loss occurred in BG3–30. This mass reduction is primarily attributed to the release of physically adsorbed water and evaporable pore water within the geopolymer matrix.^52^ At 300 °C, dehydration processes are expected to dominate the thermal response, while extensive structural decomposition of the aluminosilicate network remains limited.

The observed variability in mass loss among specimens can be attributed mainly to differences in internal pore structure, initial moisture content, and microstructural heterogeneity.^49^ Specimens exhibiting higher mass loss likely possessed greater accessible porosity or retained higher levels of evaporable water prior to heating. Conversely, lower mass loss values indicate a denser matrix or reduced moisture retention capacity. These findings are consistent with the well-established thermal behavior of geopolymer materials, in which moderate temperature exposure predominantly results in moisture release rather than severe matrix degradation. Recent investigations on various geopolymer mortars have similarly demonstrated that while geopolymer matrices exhibit excellent high-temperature stability, their thermal degradation, mass loss, and residual mechanical properties are highly dependent on the precursor types, aggregate characteristics, and specific exposure conditions.^14–16^

Overall, the combined mechanical and thermal evaluation indicates that the performance of granodiorite-modified geopolymer systems can be effectively tailored through the appropriate selection of particle size and sand content. While mechanical behavior under ambient conditions is governed by load-bearing efficiency and crack-control mechanisms, as demonstrated in Figure 5, the relatively limited mass loss observed after exposure to 300 °C confirms that these microstructural modifications do not compromise thermal stability. The developed geopolymer blocks exhibit adequate resistance to moderate thermal exposure, suggesting that the optimized compositions provide a favorable balance between mechanical performance and thermal durability, which is a critical requirement for construction-related applications subjected to temperature fluctuations.

The water absorption behavior of both granodiorite sand fractions and the corresponding geopolymer mortars was evaluated to elucidate the influence of particle size on pore structure development. The water absorption values of the granodiorite sand fractions were determined as 14.68%, 13.98%, and 12.23% for particle size ranges of <0.25 mm, 0.25–0.5 mm, and 0.5–1.0 mm, respectively. The observed decrease in water absorption with increasing particle size is attributed to the reduction in specific surface area and associated surface-bound moisture. In contrast, the geopolymer mortars exhibited an opposite trend. The water absorption values of BG1–30, BG2–30, and BG3–30 were measured as 11.27%, 14.17%, and 15.78%, respectively, indicating a progressive increase with increasing granodiorite particle size. Consistent with this trend, the bulk densities of the same specimens were determined as 1.68, 1.59, and 1.52 g/cm^3^, respectively, showing a gradual decrease with increasing particle size. This inverse relationship between density and water absorption clearly indicates that the pore structure of the geopolymer matrix becomes more open and interconnected as the granodiorite particle size increases.

The divergence between the intrinsic water absorption behavior of the sand fractions and that of the geopolymer mortars highlights that the overall fluid uptake of the mortar system is governed primarily by its microstructural organization rather than the inherent properties of the aggregate alone. The lower water absorption and higher density observed in systems containing finer particles can be attributed to improved packing efficiency and enhanced matrix densification, which reduce pore connectivity and limit water ingress. Conversely, the incorporation of coarser particles leads to increased interparticle voids and weaker matrix continuity, promoting the formation of more accessible and interconnected pore networks.

Similar relationships between aggregate characteristics, porosity, density, and resulting material performance have been reported in geopolymer systems.^21,29^ These findings are also consistent with the mechanical and thermal results presented in this study. While finer particles contribute to reduced water absorption and improved thermal insulation due to increased microstructural compactness, coarser particles enhance mechanical strength through load-bearing effects but at the expense of increased porosity. The results confirm that particle size plays a critical role in governing the balance between density, porosity, water absorption, and performance in granodiorite-modified geopolymer systems.

### 3.3 Thermal Insulation Performance of Plate-Type Geopolymer Mortars under Flame Exposure

The thermal insulation performance of the geopolymer plates (10 cm × 10 cm) was comparatively evaluated under direct flame impingement using a gas burner, reaching a steady surface temperature of approximately 700 °C (Figure S1). This configuration represents a high heat flux environment, estimated to be on the order of ∼90 kW/m^2^. While this value is provided as a theoretical estimation rather than a directly measured parameter due to the absence of a heat flux sensor, it serves to illustrate the severity of the thermal loading. During the experiments, the temperature evolution on both the front and back surfaces was continuously monitored. The exposure was maintained for 3–4 min until the back surface reached a steady-state maximum, ensuring consistent thermal loading across all compositions. It is important to clarify that this test was not intended to replicate standardized fire resistance methods, such as ISO 834 (ISO, 2012).^53^ Instead, it aimed to provide a comparative assessment of heat transfer behavior under severe, direct-flame conditions, a methodology previously utilized in the literature for geopolymer characterization.^38^

The maximum temperatures measured on the back surfaces were 93 °C for *x* <0.25 mm, 103 °C for 0.25–0.5 mm, and 207 °C for 0.5–1.0 mm fractions. These findings clearly indicate that heat transfer is significantly accelerated with increasing particle size, leading to a corresponding reduction in thermal insulation efficiency. This trend aligns with recent studies demonstrating that finer particles and the associated increase in matrix microporosity effectively restrict heat transfer, thereby enhancing the thermal insulation properties of geopolymer composites.^54^

The thermal stability and insulation performance of the mortars are further corroborated by microstructural evaluations. As observed in the SEM analysis (Figure 5), the incorporation of granodiorite modifies how the geopolymer matrix manages thermal flux. Rather than relying solely on the mass loss associated with dehydration, the mortar’s thermal resistance is governed by two complementary mechanisms: The dense, rigid granodiorite particles act as a physical heat-shielding phase that helps maintain overall dimensional integrity, while the intrinsic microporosity of the geopolymer binder effectively restricts heat transfer. This microstructural configuration prevents rapid thermal penetration, aligning with recent studies that highlight the thermal barrier effect of rigid mineral fillers within porous aluminosilicate networks.^7,47^

This observed trend, when evaluated together with the mechanical strength results of block-type (BG-coded) specimens presented in Figure 6 and the limited mass loss values reported after exposure to 300 °C in Table 3, reveals that the microstructure of granodiorite-modified geopolymer systems simultaneously governs both mechanical and thermal performance. In systems containing fine granodiorite particles, increased microporosity and air entrapment reduce the effective thermal conductivity, thereby limiting the temperature transmitted to the back surface; however, these same microstructural characteristics also restrict the load-bearing capacity, resulting in relatively lower compressive strength. In contrast, geopolymer systems incorporating coarse granodiorite particles exhibit a denser and more continuous matrix structure, which is consistent with the higher compressive strengths observed in Figure 6, but leads to a reduced thermal barrier effect.

Overall, it can be concluded that an inverse yet predictable balance exists between thermal and mechanical performance in granodiorite-modified geopolymer plates. The results demonstrate that decreasing particle size quantitatively enhances thermal insulation performance, albeit at the expense of mechanical strength, whereas coarser particles improve mechanical strength while limiting thermal insulation efficiency. These findings highlight that geopolymer systems can be rationally optimized in terms of granodiorite particle size and content according to the requirements of the intended application.

## 4 Conclusion

This study provides a comprehensive understanding of how granodiorite sand particle size and content cooperatively govern the mechanical and thermal performance of metakaolin-based geopolymer mortars through a geometry-sensitive approach. A primary finding of this research is the critical role of microstructural organization in determining the physical properties of the matrix. We observed an inverse relationship between particle size and densification: finer granodiorite particles (<0.25 mm) significantly improve packing efficiency, resulting in a higher bulk density of 1.68 g/cm^3^ and a reduced water absorption of 11.27%. In contrast, coarser particles lead to a more open and interconnected pore network, which directly influences the trade-off between load-bearing capacity and thermal resistance.

The mechanical response of the developed geopolymers is heavily dictated by the type of loading and the internal skeletal structure formed by the aggregates. Coarse granodiorite particles (0.5–1.0 mm) at moderate contents (30 wt %) were found to be most effective for block-type applications, providing a robust load-bearing framework that achieved compressive strengths as high as 67.9 MPa. Conversely, flexural performance proved to be more sensitive to matrix continuity and the characteristics of the Interfacial Transition Zone (ITZ). In this context, finer particles act as a beneficial microfiller, enhancing the density of the geopolymer gel and providing a more balanced resistance to crack initiation.

Thermal evaluation under both moderate exposure (300 °C) and intense flame loading (700 °C) further highlights the versatility of granodiorite-modified systems. While all compositions demonstrated adequate thermal stability with limited mass loss (ranging from 3.4% to 9.1%), the particle size emerged as the decisive factor for insulation performance. Plate-type specimens incorporating fine particles achieved exceptional thermal protection by reducing back-surface temperatures to just 93 °C, a behavior attributed to the increased microporosity and effective air entrapment. Ultimately, these findings establish a rational design framework, confirming that granodiorite content and size can be precisely tailored to meet the specific mechanical or thermal requirements of modern, sustainable construction applications

## Supplementary Material



## Notes

Experimental setup for flame-gun induced thermal exposure and continuous monitoring of front and back surface temperatures using a data logger device (Figure S1); high-temperature muffle furnace employed for controlled thermal exposure of geopolymer specimens (Figure S2); and step-by-step water absorption testing procedure including oven-drying and immersion stages for both geopolymer mortars and granodiorite sand fractions (Figure S3) (PDF)
