# Supplementary material for: Effect of Granodiorite Sand Content and Particle Size on the Mechanical and Thermal Performance of Metakaolin-Based Geopolymer Mortar
Source: ACS Omega. 2026 Jun 17;11(25):37346–56. doi: 10.1021/acsomega.6c01784 (PMC13325165; doi:10.1021/acsomega.6c01784)
Supplement: Supplementary file 1 [file ao6c01784_si_001.pdf]

Effect of Granodiorite Sand Content and Particle Size on the Mechanical and Thermal Performance of Metakaolin-Based Geopolymer Mortar

*Yildiz Yildirim[a], Gurkan Akarken[b,c], Gokçe Kayatepe[b], Ugur Cengiz[c,d]**

[a]*Kale Ceramic R&D Department, Canakkale, Turkey*

[b]*Department of Energy Resources and Management, Faculty of Engineering, Çanakkale Onsekiz Mart University, Çanakkale, Türkiye*

[c]*AFC Green Technologies R&D, Canakkale Technopark, Sarıcaeli, 17100, Çanakkale, Turkiye*

[d]*Surface Science Research Laboratory, Department of Chemical Engineering, Faculty of Engineering, Çanakkale Onsekiz Mart University, Çanakkale, Türkiye*

*Corresponding author: ucengiz@comu.edu.tr

Phone: +90 (286) 218 00 18

Supporting Information (SI)

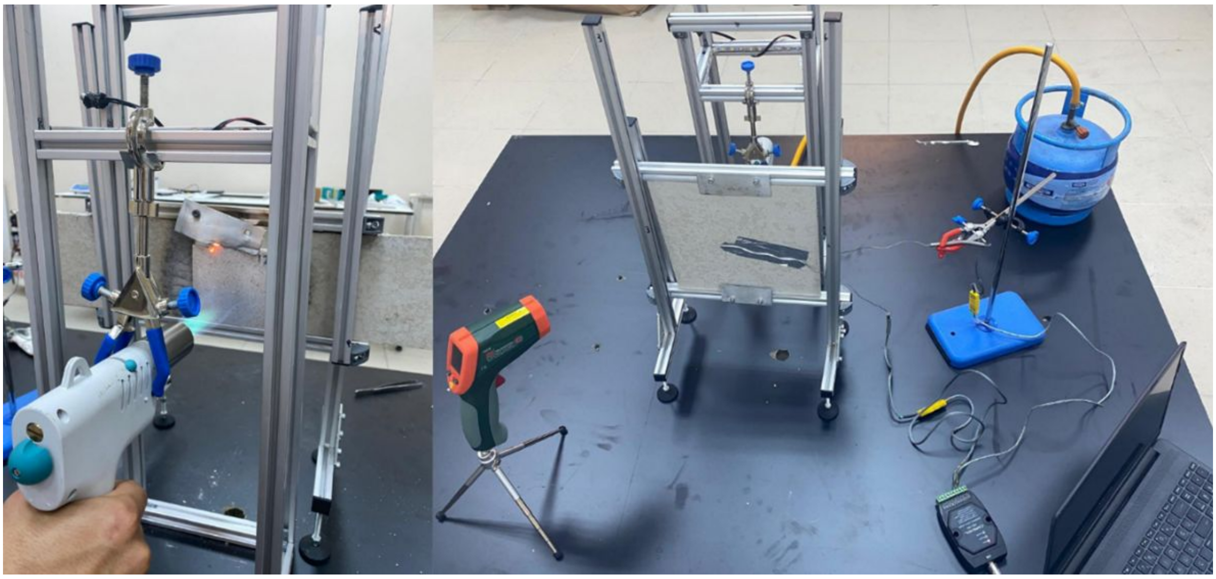

Figure S1. Experimental setup for flame gun induced thermal exposure and continuous monitoring of front and back surface temperatures using a data logger device.

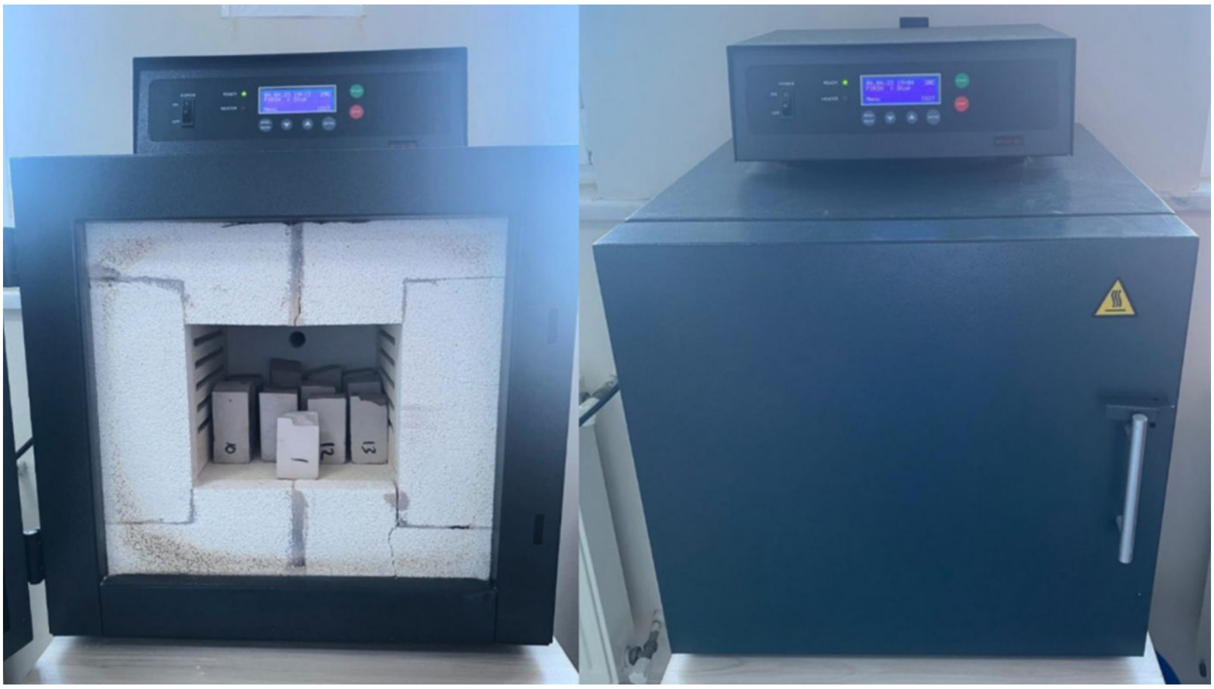

Figure S2. High-temperature muffle furnace employed for thermal exposure of geopolymer specimens at controlled heating conditions.

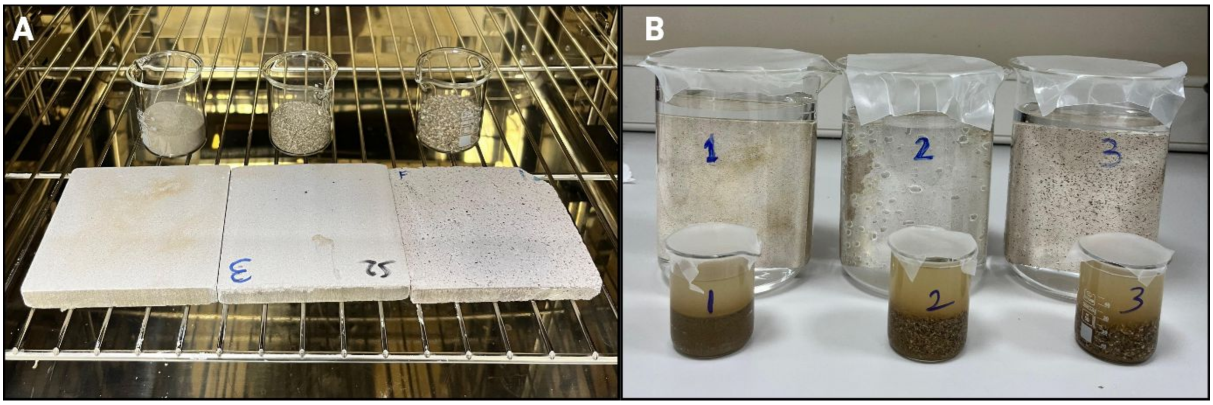

Figure S3. Water absorption testing procedure for granodiorite-modified geopolymer specimens and sand fractions. (A) Oven-drying of geopolymer plates and granodiorite sand fractions prior to testing. (B) Immersion of samples in water for 24 h to achieve saturation, followed by removal of excess surface moisture before mass measurements.
